# Calf Morbidity and Mortality: Critical Challenges for Smallholder Dairy Farmers in Northern Ethiopia

**DOI:** 10.1155/vmi/2388659

**Published:** 2025-03-26

**Authors:** Gebreyohans Gebru, Gebregergs Tesfamaryam, Dawit Gebremichael, Gebremedhin Romha, Angesom Hadush, Tsriti Gebremeskel, Kiros Kelkay, Moges Gebremichael, Alem Beyene, Haftom Hadush

**Affiliations:** ^1^Department of Animal Science, College of Agriculture, Aksum University, Aksum, Ethiopia; ^2^Department of Veterinary Public Health and Food Safety, College of Veterinary Sciences, Mekelle University, Mekelle, Ethiopia; ^3^Department of Agro-Economics, College of Agriculture, Aksum University, Aksum, Ethiopia; ^4^Department of Medical Laboratory, College of Health Sciences, Aksum University Referral Hospital, Aksum, Ethiopia

**Keywords:** calf morbidity, calf mortality, Northern Ethiopia, risk factors

## Abstract

Calf morbidity and mortality pose significant economic challenges for smallholder dairy farms in Ethiopia, resulting in direct losses from calf deaths, replacement costs, treatment expenses, and reduced lifetime productivity. This study aimed to comprehensively investigate the magnitude and epidemiological characteristics of calf morbidity and mortality in Northern Ethiopia. A cross-sectional study with mixed approaches was carried out from December 2019 to September 2020. A total of 183 questionnaire survey, four focus group discussion (FGD), and 17 key informant interviews (KII) were included in the study. Furthermore, participatory epidemiological appraisals were incorporated to triangulate and strengthen survey evidences. Analysis of survey results revealed that 69.4% of the farmers have experienced calf morbidity, while 63.9% of them have encountered calf mortality. Similarly, results of proportional piling indicated that calf morbidity and mortality were estimated to occur in 75.5% and 55.9% of the farms, respectively. Moreover, all KIIs had encountered calf morbidity, while 88.2% of them had faced calf mortality. Ninety percent of KIIs, 66.2% of the participants of community-based epidemiology, and 27.87% of questionnaire survey respondents suggested that calf morbidity and mortality occur in less than one-week-aged calves. Regarding the potential risk factors, source of water, frequency of barn disinfection, breed types, health status of dams, using separate calf housing, amount of colostrum provided to calves, and cleaning frequency of barns had statistically significant association with the occurrence of calf morbidity and mortality (*p* < 0.05). Additionally, results of participatory appraisal, FGDs and KIIs showed that calf diarrhea, nutritional disorder, pneumonia, and navel ill were the leading causes of calf morbidity and mortality. Furthermore, observation assessment showed that most dairy farms were surrounded by dense human settlements, livestock markets, and municipal slaughtering houses. Hence, the farms had critical space limitation (for animals to exercise) as well as poor drainage systems and hygienic practices. Our assessment also showed that lack of veterinary services, shortage of water supply, and poor artificial insemination services were the major challenges of dairying in the area. In conclusion, the present study revealed that calf morbidity and mortality were critical challenges for dairying in Northern Ethiopia. Furthermore, the study highlighted the epidemiological characteristics and potential risk factors associated with calf morbidity and mortality, awareness gaps in calf management, as well as key bottlenecks in dairy farming. These findings underscore the need for a comprehensive study, continuous capacity building initiatives, improved infrastructure, and services to mitigate calf losses.

## 1. Background

Livestock is one of the most important assets and resources for many poor farmers around the world [1], including Ethiopia [2]. Livestock contribute more than just food: They also help with tasks like plowing fields, provide income in tough times, and offer valuable products like leather, fuel, and fertilizer, and in places where financial institutions are lacking, livestock can even be used as a form of savings [3]. For millions of poor farmers, livestock is more than just a resource. It is a part of their social and cultural lives. Owning animals helps them farm sustainably and ensures their financial security [4, 5]. Sub-Saharan African countries like Ethiopia largely depend on livestock production. Ethiopia is estimated to have about 59.2 million cattle, including 10.5 million dairy cows. These cows produce around 3.6 billion liters of milk annually [6]. Dairying is growing in importance, and calves are crucial for the future of dairy farms [3, 7, 8]. The government is focusing on developing the dairy industry to increase milk production from small farms [9, 10]. Many developing countries, like Ethiopia, rely on small farms for dairy production. This provides essential nutrition and income for millions of families [11]. The demand for milk and dairy products in urban and peri-urban areas has surged dramatically, with national per capita consumption reaching a substantial 27 L annually [12]. However, to meet the rapidly growing demand for milk and dairy products, a significant increase in the number of high-yielding, well-adapted cows is imperative [13]. Given the substantial potential for smallholder income and employment growth through high-value dairy products [14], developing Ethiopia's dairy sector can significantly enhance food and nutrition security, thereby contributing to poverty reduction in the country. The success of any breeding program and the future viability of smallholder dairy farms pivots on the survival rate of newborn calves.

However, calf morbidity and mortality pose a significant threat to dairy business operators, as most dairy farms grapple with acute calf health challenges [15, 16]. The health of replacement calves is a critical determinant of overall dairy operation profitability [17], as dairy heifer calves are the building blocks of future milking herds. Furthermore, high calf mortality and illness lead to significant economic losses for dairy producers due to death, treatment costs, reduced productivity, and hindered herd growth and genetic improvement [18]. Numerous studies around the world have investigated calf illness and death rates, identifying their causes and contributing factors with smallholder dairy farmers; in particular, they face alarmingly high calf mortality rates, which can go up to 50% [1]. Diarrhea in newborn calves and pneumonia in older calves are the primary causes of calf illness and death [19]. Some African countries in tropical regions have reported varying calf mortality rates. For example, in Tanzania, calf mortality rates can range from 9% to 45% [20]; in Mali, the range is between 10% and 25% [21]; and in Sudan, dairy farms in Khartoum reported a 4.9% calf mortality rate [22]. On average, 23% of Holstein Friesian female calves born at Holleta Farm in Ethiopia died before reaching their first calving age. On average, 23% of Holstein Friesian female calves born at Holleta Farm in Ethiopia died before reaching their first calving age [23]. In general, calf mortality rate in Ethiopia ranges from 7% to 30.7% [24, 25]. It is estimated that a 20% calf mortality rate can reduce net profits by 38% [26].

Calf diseases that lead to illness and death are caused by a complex interplay of factors, including management practices, environmental conditions, the calf's mother, and the calf itself [27]. It is obvious that the housing conditions of livestock can greatly affect their health and productivity [15]. The combination of a compromised immune system and a lack of prior exposure to infection renders newborn calves highly susceptible to infectious diseases [27]. Passive immunity can be achieved through the ingestion of adequate volumes of colostrum by calves. Other factors impacting calf health and survival of calves include herd production levels, antibiotic use, weaning age, and calf separation or mixing [28, 29].

In Ethiopia, the morbidity and mortality recorded by different authors from different parts of the country were 66.7% and 20% [30], 62.0% and 22.0% [15], 29.3% and 9.3% [24], 58.4% and 30.7% [25], and 30.9% and 8.64% [31], respectively. The primary causes of calf morbidity and mortality include calf diarrhea, calf pneumonia, navel ill (omphalitis), septicemia, vector borne diseases and helminthes, and nutritional problems [32]. Similarly, Fentie et al. [33], Tora et al. [34], and Ahmedin and Assen [35] have reported that diarrhea and pneumonia (respiratory disorder) were the most common causes of calf morbidity and mortality in that order. Like in other areas of Ethiopia, calf morbidity and mortality are serious challenges for small holder dairy husbandry in Tigray regional state. The frequent occurrence of diseases, significant calf losses, and the ineffectiveness of routine treatments have driven many farmers to financial ruin [36]. These, coupled with the absence of record-keeping, are the major challenges in dairy husbandry and milk production in the area (personal observation). Most importantly, prior assessments have not been conducted, and calf morbidity and mortality in this region remain poorly documented. Therefore, this study aimed to provide preliminary evidence on magnitude and epidemiological characteristics of calf morbidity and mortality in Northwestern and Central zones of Tigray that can be used to develop and apply cost-effective prevention and control measures.

## 2. Methodology

### 2.1. Description of Study Area

**Figure 1:**
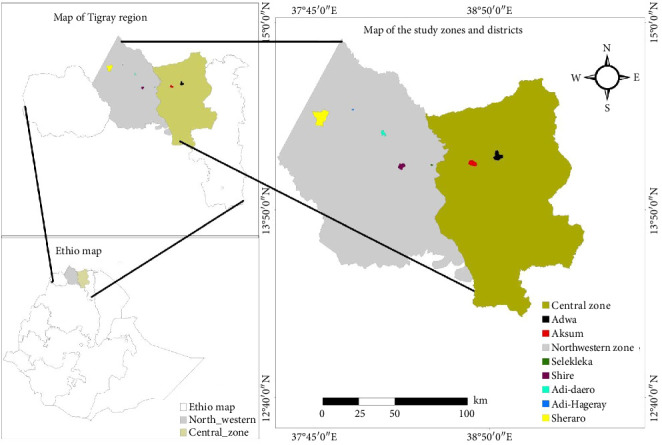
Map of the study area.

The study was conducted in Central and Northwestern zones of Tigray, Ethiopia (Figure 1). Central zone of Tigray is located about 963 kms away to the North from Addis Ababa. The geographical coordinates are 14° 7′ 8″ N, 38° 43′ 46″ E with altitude range of 2000 to 3000 m above sea level (masl). The average daily temperature is 18.3°C with average annual rainfall of 861 mm. There were 446 dairy farms with 2189 cows in Central zone. Similarly, Northwestern Tigray is a zonal administration located at about 1126 kms away to the North of Addis Ababa and its altitude ranges from 645 to 2852 masl and its coordinates are 14°01′13.4″N, 38°09′50.0″E. The annual temperature of the area varies from 18°C to 34.6°C. The annual rainfall of Northwestern zone of Tigray ranges between 450 and 800 mm. In Northwestern zone, there were 374 dairy farms with 1687 dairy cows. Overall, there were 820 dairy farms and 3876 dairy cows in both zonal administrations. Both Central and Northwestern zones are assumed to have relatively good potential for crop and livestock production [6].

### 2.2. Study Design

A cross-sectional study was carried out to assess calf morbidity and mortality from December 2019 to September 2020. Both quantitative and qualitative data collection tools were used as described by Creswell [37]. These will include semistructured questionnaire surveys, KII, FGDs, participatory epidemiological approach, and field observation. Smallholder dairy farms with five or more years of farming experience in urban and peri-urban settings were included in our study.

### 2.3. Sampling and Sample Size Determination

A total of seven woredas were included in this study. Two woredas from Central zone (Aksum and Adwa towns) and five woredas from Northwestern zone (Selekleka, Shire, Sheraro, Adi-hageray, and Adi-daero) were included in this study purposively based on abundance of dairy farms and their access to road transport. From each woreda, sample households were selected using simple random sampling method. According to the information obtained from the Northwest and Central agriculture office, there were nearly 900 dairy farms operated by individuals and cooperatives, and we included 20% of the dairy farms in our assessment according to Kothari [38]. Therefore, 183 dairy farms were considered in the household interviews to assess the knowledge, attitude, and practices (KAPs) of smallholder farms. In addition, we included data of four FGD comprising 8–10 persons of different sex. Additionally, 17 KII comprising veterinarians, animal husbandry experts, and artificial insemination technicians were included in our study, and the methods were adopted from Hennink et al. [39] and Muellmann et al. [40]. Furthermore, four participatory discussion groups (community-based epidemiological studies) were incorporated to include further evidences.

### 2.4. Data Collection Methods

Prior to the actual data collection, a preliminary survey was conducted to identify the dairy cattle potential, evaluate the extent and distribution of calf morbidity and mortality, and make logistical arrangements. Then, quantitative and qualitative data were collected using questionnaire survey, FGD, KIIs, participatory appraisal, and observational study methods.

#### 2.4.1. Quantitative Data

A semistructured questionnaire survey was prepared and administered to farmers or farm attendants to evaluate the history of experiencing calf morbidity and mortality and assess the potential risk factors and overall management aspects based on their KAPs. With this method, the KAPs of farm owners, animal-related factors (age, breed, and sex), and environmental factors (management, feeding, type of feed, and hygiene practices) were assessed. The questionnaire was pretested on field to amend potential errors and validate it.

#### 2.4.2. Qualitative Data

Key informant interviews (KIIs): KIIs comprising veterinarians, artificial inseminators, and animal production experts were included in our study to pinpoint the potential risk factors and causes of calf morbidity and mortality, extent of the problem, any diagnostic trials conducted, and treatment pathway as well as the suspected causes of the problem. Nonstructured questionnaires were prepared and provided to key informants. The key informants recorded their responses on the free spaces provided with script.

Focus group discussion (FGDs): FGDs were conducted to collect consensus-based views and suggestions of participants regarding the extent of the problem, probable causes, and risk factors of calf morbidity and mortality. Four FGDs comprising 8–10 persons of different sex and age were conducted. A set of open-ended questions were prepared, and FGD participants were asked by the moderator to speak openly their views and comments. The responses of each FGD participant were recorded by the taker word-for-word with legible handwritings.

Participatory epidemiology: Participatory epidemiological appraisal methods such as pairwise ranking, matrix scoring, proportional piling, and seasonal calendar were employed to prioritize the probable causes and characterize the epidemiological characteristics of calf morbidity and mortality in the area. The participatory epidemiological appraisals were set to hold 8–10 adult participants of heterogeneous sex and conducted as described by Catley [41].

Observational study: A checklist of questions was used to assess the hygienic condition, space allocation per animal, nutritional status of herds, and relative location of the farms as described by Gebru and Gebretinsae [42]. Using personal observation, pertinent data were recorded by handwritten notes and supported by photographs for visual analysis.

### 2.5. Data Management and Analysis

All data collected were entered and coded in Microsoft Excel spreadsheets and analyzed using SPSS-20 statistical software. Descriptive statistical analyses were employed to summarize the results. Chi-square was used to test the statistical significance of independent variables. Confidence level was considered at 95% and *p* ≤ 0.05 as level of significance. The qualitative data were analyzed using thematic analysis approach as described by Graneheim and Lundman [43].

## 3. Results

The result section comprises household surveys of KAP assessment on calf morbidity and mortality, FGD, and KII complemented by participatory epidemiological evidences. The result of each component is presented as follows:

### 3.1. Analysis of Household Survey

#### 3.1.1. Potential Risk Factors of Calf Morbidity

About 69.4% of the farmers have experienced calf morbidity in the preceding 5 years. Further, study zones and districts as well as health problems of dams were statistically significant. Although it was statistically insignificant, most cases of calf morbidity (27.9%) occur in the first week age of claves. Similarly, 41% of the respondents stated that calf morbidity does not depend on season (Table 1). Calf diarrhea, nutritional disorder, navel ill, and pneumonia were the most blamed causes of calf morbidity.

#### 3.1.2. Potential Risk Factors of Calf Mortality

About 63.9% of the farmers have experienced calf mortality in the preceding 5 years. With regard to analysis of potential risk factor, study zones and districts, health problems of dams, sex of dairy owners, and type of breed were statistically significant. Although it lacks statistical significance, most incidents of calf mortality (52.7%) do not depend on season (Table 2). Calf diarrhea, nutritional disorder, navel ill, and pneumonia were the leading causes of calf mortality.

#### 3.1.3. Management Practices and Calf Morbidity

In terms of farming practice, source of water, frequency of disinfection of barns, using separated housing of calves from the rest of the herd, as well as the amount of colostrum provided to calves had statistically significant association with the occurrence of calf morbidity (Table 3).

#### 3.1.4. Management Practices and Calf Mortality

Source of water, frequency daily cleaning of barns, and using separated housing of calves from adult cows had statistically significant association with the occurrence of calf mortality in the study area (Table 4).

### 3.2. FGD

To complement the household surveys with further supporting evidences, we incorporated four FGD assessments. Most FGD participants stated that they experienced calf morbidity and mortality. Although some participants argued calf morbidity and mortality mainly occur during rainy seasons, the FGD participants concluded that they appear throughout the year regardless of the seasons. FGD participants also suggested that most cases of calf morbidity and mortality occur mainly in one-week-aged calves. FGD participants stated that they do not have any clues on the risk factors and probable causes of calf morbidity and mortality, but considerable farmers believe that poor hygiene could be a potential risk factor. Watery diarrhea, coughing, poor appetite, and swelling around the umbilicus were reported to be the most commonly observed clinical signs during the FGD approach.

### 3.3. KIIs

We also included 17 key informants in our study. The professions of the key informants were veterinarians (64.7%), AI technicians (23.5%), and others (11.8%) including animal production experts. All the key informants have experienced calf morbidity during their service delivery and supervision activities. Similarly, the majority (88.2%) of the key informants have faced calf mortality in their working areas. Almost half of the key informants (47.1%) specified that the occurrence of calf morbidity and mortality does not depend on the season. On the other hand, 29.4% and 23.5% of the key informants explained that the occurrence of calf morbidity and mortality occurs during winter/dry season and summer/wet season, respectively. All of the key informants supposed that greater than 90% of the calves faced morbidity and mortality within less than 1 week. All key informants explained that the most risk factors of calf morbidity and mortality were poor hygienic and sanitation practices, poor drainage systems, and inappropriate location of the farms. According to the key informants, the main causes of calf morbidity and mortality were calf diarrhea, pneumonia, and nutritional deficiency.

### 3.4. Participatory Epidemiology

The findings of participatory epidemiology were summarized as follows:

#### 3.4.1. Simple Ranking

Using simple ranking approach, calf diarrhea, nutritional disorder, pneumonia, calf blindness, epilepsy, and naval ill were the top most prevalent diseases and causes of mortality in dairy farms in Northwestern areas while lack veterinary services and limitations of drug supply; in appropriate site selection; lack of drainage; insufficient space of farms (to accommodate large herd size with no space for excising); and lack of water supply were the major challenges of dairy farms in the study area. However, diarrhea, manges (ectoparasites), pneumonia, naval disease, and blindness were the most common health problems of calves in Central zone, mainly Axum area; shortage of feed, poor animal health service, limitation of farming spaces, poor AI service, lack of market linkage, and shortage of water supply were prioritized as top challenges in dairy farms.

#### 3.4.2. Pairwise Ranking

**Figure 2:**
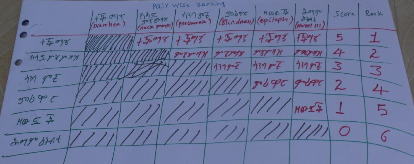
Pairwise ranking of common causes of calf morbidity and mortality.

Pairwise assessment result indicated that diarrhea, nutritional disorder, pneumonia, calf blindness, epilepsy, and naval ill were the top ranked six causes of calf morbidity and mortality in Northwestern Tigray (Figure 2), while diarrhea, naval disease, pneumonia, ectoparasites, and blindness syndrome were common health problems in Central zone.

#### 3.4.3. Proportional Piling

**Figure 3:**
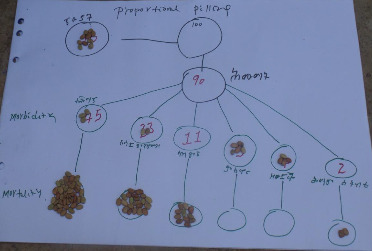
Proportional piling result of Northwestern Tigray.

**Figure 4:**
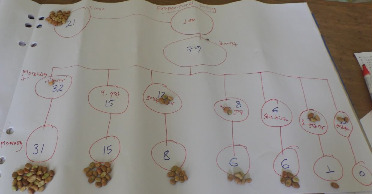
Proportional piling result of Central Tigray.

Using a proportional piling approach, we provided 100 beans (equivalent to 100 calves) to the participants to assign the morbidity and mortality rates among newborn calves. Accordingly, the overall prevalence of calf morbidity and mortality were estimated to be 75.5% and 55.9%, respectively. With regard to occurrence of the diseases among different zones, the average morbidity rates were 82% in Northwestern zone (Figure 3) and 79% in Central zone (Figure 4), while the mortality rates were estimated to be 69.8% in Northwestern zone and 42% in Central zone of region, respectively. Moreover, proportional piling method was employed to estimate the average morbidity rates calves by age and recorded as follows: 66.2 in one-week-old calves; 14.8% in 1- to 2-week-old; 7.6% in 2 to 4-week-old; 5.1% in 4 to 12–week-old; 3.8% in 12 to 24-week-old; and 2.7% in and 24 to 52-week-old calves. Average proportional piling results of mortality rates by age were 86.8%, 70.9%, 51.5%, 46.7%, 50%, and 12.5% of less than 1 week, 1-2 weeks, 2–4 weeks, 4–12 weeks, 12–24 weeks, and 24–52 weeks, respectively.

#### 3.4.4. Matrix Scoring

Thirty beans were provided to participants to identify the indicators of calf diarrhea, paralysis or nutritional disorder, pneumonia, navel infection, and calf epilepsy. The corresponding results revealed that coughing and weight loss were 100% indicators for pneumonia and calf diarrhea, respectively. About 90% of calf diarrhea and 10% of navel infection showed watery, greenish, or profuse diarrhea. The participants were also indicated that recumbence was an indicator for nutritional disorder at 66.7%, and 33.3% of calf epilepsy. And, 100% of navel infection showed swelling and abscess/pus around the umbilicus. About 86.7% of pneumonia and 13.3% of calf diarrhea cases showed nasal discharges. Lacrimation was an indicator for pneumonia at 80%, nutritional disorder at 13.3%, and calf diarrhea at 6.7%.

#### 3.4.5. Seasonal Calendars

**Figure 5:**
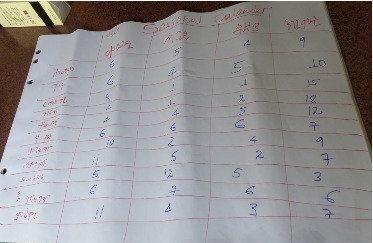
Seasonal occurrence of calf morbidity and mortality.

Further, 30 beans were set to assess the seasonal pattern of calf morbidity and mortality rates using proportional piling approach. Accordingly, calf morbidity was estimated to occur in autumn, winter, spring, and summer with average rates of 20.8%, 21.8%, 24.7%, and 30%, respectively. In Northwestern zone, calf morbidity occurred in autumn, winter, spring, and summer seasons with average rates of 20%, 22%, 28% and 30%, respectively, while 27%, 21.6%, 21.4%, and 30% occurred in autumn, winter, spring, and summer seasons with average rates of 20%, 22%, 28%, and 30%, respectively, in Central zone (Figure 5).

### 3.5. Personal Observation

During the personal observation, most dairy farms have stagnated liquid wastes in their compound. This suggests the farms to have poor drainage setups. Besides, the farms are characterized to have poor hygiene and sanitation practices. Most importantly, the significant farms were concentrated in locations with dense human settlements and in close proximity to open livestock markets, which could lead to spillover infection to human residents (zoonosis) and dairy cows (reverse zoonosis). Likewise, our personal observation revealed that some farms were found closer to municipal abattoirs, where the abattoirs themselves do not have secure fences (pet proof).

## 4. Discussion

The present study revealed that calf morbidity and mortality were among the serious challenge for dairying in the study area. About 69.4% and 63.9% of the questionnaire respondents had experienced calf morbidity and mortality, respectively, during the preceding 5 years. These proportions were higher than previously reported studies in Ethiopia [44]. This might be due the difference in study design, agro-ecological settings, management practices, as well as availability infrastructure and service in the study areas.

According to the questionnaire survey, calf diarrhea was the primary cause of both morbidity and mortality, accounting for 43.8% and 33.3%, respectively. This finding aligns with the reports of Ahmedin and Assen [35], Tesfaye et al. [44], and Alemu et al. [45]. However, our finding was lower than that of reported by Abebe et al. [46], but it was higher than the reports of Tesfaye et al. [44] and Megersa et al. [24]. The difference might be due to varying farming practices. The higher prevalence of diarrhea in our study may be linked to poorer hygiene, sanitation, and colostrum feeding habits on the farms. For example, the respondents believe that colostrum can harm young calves. Qualitative results such as FGDs, KII, and participatory epidemiology, and qualitative studies revealed that nutritional disorders, navel ill, and pneumonia were other leading causes of morbidity and mortality which required comprehensive investigation and targeted intervention. Particularly, both quantitative and qualitative studies were the same in identifying diarrhea as the primary cause of morbidity and mortality. Similar results were reported from different areas of the country in identifying diarrhea as the primary cause of morbidity and mortality. Similarly, this was reported from different areas of the country by other authors: Ahmedin and Assen [35], Tesfaye et al. [44], Alemu et al. [45], Abebe et al. [46], and Megersa et al. [24] though the specific rates for each cause varied. Diarrhea and pneumonia were the blamed causes as reported by Wudu et al. [15], Fentie et al. [33], Ferede [25], Asmare and Kiros [47], and Dagne et al. [48]. Nutritional disorders also pose a significant health threat to dairy farms, necessitating a comprehensive investigation and targeted intervention. Indeed, Mohammed et al. [49] reported on most of these disease conditions that most of these disease conditions were also identified as major health problems in dairy farms, next to diarrhea. Pneumonia was identified as a cause of both morbidity and mortality, although at a lower rate than reported by Ahmedin and Assen [35], Alemu et al. [45], and Abebe et al. [46]. Furthermore, our personal observations confirmed that most farms were established in flat areas leading to poor drainage systems and stagnated liquid wastes in their compounds and were characterized to have poor hygiene and sanitation practices. It is obvious that poor hygiene and sanitation can lead to calf morbidities and mortalities in farms. Significant farms were also found concentrated in locations with dense human settlements and in close proximity to open livestock markets and municipal abattoirs, which could lead to spillover infection to human residents (zoonosis) and dairy cows (reverse zoonosis). This suggests the farms were established for short-term plans without careful consideration of expansion plans (master plans) of the respective towns.

Although it was statistically insignificant (*p* > 0.05) in the household survey, all key informants claimed that more than 90% of the cases of morbidity and mortality occur in less than one-week-aged calves. Similarly, FGD participants also suggested that most cases of calf morbidity and mortality occur mainly in the first week after birth. Results of participatory epidemiology are also similar to findings of KII and FGD that large numbers of cases occur in one-week-aged calves, followed by 1-2 weeks aged. Similar findings on calf morbidity and mortality at the early ages have been reported from different studies in Ethiopian such as Wudu et al. [15], Tesfaye et al. [44], Abebe et al. [46], Ferede et al. [25], and Mohammed et al. [49], as well as other studies conducted in Sweden [19] and Norway [50]. These early-age morbidity and mortality rates could be linked to poor calf management practices, including inadequate farm sanitation, insufficient colostrum feeding, and failure to vaccinate dams. Most importantly, farmers who attended the participatory epidemiology and FGD assessments claimed that most diseased calves did not respond to conventional antibiotic therapies. Furthermore, seasonal pattern assessment toward the occurrence of calf morbidity and mortality was also evaluated. The results showed that both calf morbidity and mortality had no significant difference (*p* > 0.05) across the seasons of the year. The findings of the FGD and KII results were also confirmed that calf morbidity and mortality had no distinct seasonal pattern.

Among the hypothesized risk factors, study zones/districts, sex of dairy owners, type of breeds, and health problems of dams were significantly associated (*p* < 0.05) with calf morbidity and mortality. Northwestern zone experienced 2.48 times higher calf morbidity and mortality rates than those of Central zone. This could also be explained by the level of animal health intervention and awareness creation efforts by governmental and nongovernmental entities or agroecological characteristics of the areas. Regarding the sex of dairy farm owners, male owners experienced twice higher calf morbidity and mortality rates than female farm owners. This could be attributed to women's generally better adherence to hygiene and sanitation practices on farms compared to men. Females are highly effective and efficient as primary contributors to livestock husbandry practices. Similar finding was reported by Belay and Mekibib [51] that calf mortality rates were significantly lower on female-managed farms. With regard to breed types, crossbred calves were significantly more susceptible to morbidity and mortality compared to local breed calves. Alemu et al. [45] had reported similar findings, that local breeds exhibit greater tolerance to infections compared to crossbred calves. Calves born to dams having health problems were more at risks of morbidity and mortality. Yitagesu et al. [52] reported a similar finding that calves born to dams experiencing health complications, such as dystocia, were significantly associated with morbidity and mortality.

Management practices such as early calf separation, infrequent barn cleaning, inadequate disinfection, insufficient colostrum feeding, and poor water source were identified as significant risk factors for calf morbidity and mortality in this study (*p* < 0.05). Moreover, farm owners who failed to separate calves immediately from their dams and not disinfect their barns experienced higher morbidity and mortality rates than farm owners who implemented separating of calves from the dams and disinfection practices. Previous studies reported similar results that calves stayed with their dams and kept in unclean barns were more susceptible to infections [15, 35, 51]. Regular barn disinfection can significantly reduce pathogen loads, minimizing the risk of morbidity and mortality [15].

In the present study, calves which received less than 3 L of colostrum daily were more likely to develop health issues. This was consistent with previous study that calves which did not receive 3–4 L of colostrum per day were highly susceptible to infections [51] due to the fact that colostrum is the sole source of immunoglobulins, essential for protecting neonatal calves from infectious diseases [53, 54]. As a result, inadequate colostrum feeding was significantly associated with increased calf morbidity and mortality [45]. While almost all dairy farm owners in the study area provided colostrum to their calves promptly after birth, the quantities provided were often less than the daily recommendations. Farmers had misperceptions about the quantity of colostrum who believed that large amount of colostrum is dangerous to newborn calves [53, 54]. This misperception was evident during FGDs and participatory appraisals.

Similarly, farmers used river water were more likely to experience calf diseases compared to those used tap water. Similar results were reported that providing surface water to calves significantly increases the risk of exposure to waterborne diseases [51]. Using contaminated water for drinking and cleaning milk utensils, udders, and teats poses a significant risk of microbial contamination, leading to the spread of waterborne diseases [55], because water sources are frequently contaminated with coliforms, *E. coli,* and other enteric bacteria, leading to the development of calf diarrhea [56]. Generally, the discrepancies observed in the hypothesized risk factors can be attributed to variations in breed, farm characteristics, agroecological conditions, farming objectives, and diverse management practices.

## 5. Conclusion

The study identified that calf morbidity and mortality are serious challenges for smallholder dairying. Source of water, breed type, health status of dams, frequency of barn disinfection, using separated calf housing, amount of colostrum feeding, and cleaning frequency of barns were the potential risk factors of calf morbidity and mortality. Calf diarrhea, nutritional disorder, pneumonia, and navel ill were the major causes of calf morbidity and mortality. Poor hygiene and sanitation practices were also common in the dairy farms; and most of the dairy farms were concentrated in locations with dense human settlements, livestock markets, and municipal slaughtering houses. In addition, our assessment showed that lack of veterinary services and limited drug supply, shortage of water supply, poor AI services, and space limitation for exercising animals were the major challenges of dairy farms in the study area. These suggest that continuous capacity building trainings are required to reduce the burden of calf morbidity and mortality. Improving water supply, and veterinary and AI services would be crucial for the success of the farms. Furthermore, the locations of the dairy farms should be reconsidered as per the criteria set by the ministry of urban development and housing, and at least 5 km far from dense human settlements, livestock markets, and slaughter houses.

## Figures and Tables

**Table 1:** Potential risk factors of calf morbidity in smallholder dairy farms.

Variable	Categories	Morbidity				Chi-square value	Df	Pearson (*p* value)
		Yes		No				
		Frequency	%	Frequency	%			
Zone	Northwestern	78	79.6	20	20.4	7.50	1	0.006
	Central	49	59.8	33	40.2			

District	Adwa	7	41.2	10	58.8	20.15	6	0.003
	Aksum	44	65.7	23	34.3			
	Shire	64	83.1	13	16.9			
	Selekleka	5	45.5	6	55.5			
	Adi-hageray	3	100	0	0			
	Sheraro	3	75	1	25			
	Adi-daero	4	100	0	0			

Sex of the owner	Male	94	71.8	37	28.2	0.44	1	0.5
	Female	33	67.3	16	32.6			

Age of the owner (in years)	Less than 30	30	75	10	25	1.83	2	0.4
	30–65	85	68	40	32			
	Greater than 65	6	66.7	3	33.3			

Education status of the owners	Illiterates	17	63	10	38	1.51	4	0.8
	Grade 1–4	50	72.5	19	27.5			
	Grade 5–8	32	59.3	12	40.7			
	Grade 9–12	9	64.3	5	35.7			
	Diploma+	17	63	10	38			

Age of calf	Less than 1 week	34	100	0	0	2.78	5	0.15
	1–2 weeks	22	95.7	1	25			
	2–4 weeks	32	97	1	3			
	4–12 weeks	23	100	0	0			
	12–24 weeks	8	100	0	0			
	24–52 weeks	3	75	1	25			

Seasonal occurrence of calf morbidity	Winter/dry season	39	100	0	0	4.25	2	0.13
	No depend on season	43	93.5	3	6.5			
	Summer/rain season	23	88.5	3	11.5			

Health problem of dam	Yes	27	100	0	0	15.84	2	0.001
	No	92	86.8	14	13.2			
	Do not know	5	50	5	50			

Breed	Local	8	57.1	6	42.9	2.40	2	0.3
	Crossed	120	72.7	45	27.3			
	Pure exotic	2	50	2	50			

Years of farming experience	< 5 years	65	71.4	25	28.6	2.01	4	0.7
	5–10	46	74.2	16	25.8			
	10–15	11	61.1	7	38.9			
	15–20	4	57.1	3	42.9			
	> 20	4	80	1	20			

Nature of the farm	Urban	123	70.3	52	29.7	0.8	1	0.4
	Rural	6	85.7	1	14.3			

**Table 2:** Potential risk factors of calf mortality.

Variables	Categories	Yes		No		Chi-square value	Df	*p* value
		No	%	No	%			
Zones	Northwestern	72	73.5	26	26.5	7.85	1	0.006
	Central	45	52.9	40	47.1			

Districts	Adi-daero	4	100	0	0	18.52	6	0.003
	Shire	60	77.9	17	22.1			
	Selekleka	5	45.5	6	55.5			
	Sheraro	2	50	2	50			
	Adi-hageray	2	66.7	1	33.3			
	Aksum	38	56.7	29	53.3			
	Adwa	6	35.3	11	64.7			

Sex of the owner of the farm	Male	91	67.9	43	32.1	3.44	1	0.08
	Female	26	53.1	23	47.9			

Age of the owners of the farm	Less than 30	32	68.1	15	31.9	0.20	2	0.8
	30–65	79	62.7	47	37.3			
	Greater than 65	6	60	4	40			

Education status of head	Illiterates	19	73.1	7	26.9	2.53	4	0.7
	Grade 1–4	15	55.6	12	44.4			
	Grade 5–8	44	61.1	28	38.9			
	Grade 9–12	30	68.2	14	31.8			
	Diploma+	9	64.3	5	35.7			

Age of the calves	Less than 1 week	18	100	0	0	3.52	5	0.5
	1-2 weeks	30	93.8	2	6.2			
	2–4 weeks	35	97.2	1	2.8			
	4–12 weeks	25	100	0	0			
	12–24 weeks	5	100	0	0			
	24–52 weeks	1	100	0	0			

Seasonal occurrence of calf morbidity	No dependence on season	58	96.7	2	3.3	1.41	2	0.5
	Winter/dry season	29	93.5	2				
	Summer/rain season	23	88.5	3	11.5			

Dams having any health problems	Yes	23	85.2	4	14.8	8.62	2	0.02
	No	83	78.3	23	21.7			
	Do not know	4	40	6	60			

Types of breeds	Local	5	35.7	9	64.3	7.79	2	0.02
	Crossed	111	67.3	54	32.7			
	Pure exotic	1	25	3	75			

Years of farming experience	< 5 years	57	62.6	34	37.4	1.42	4	0.9
	5–10	42	67.7	20	22.3			
	10–15	10	55.6	8	44.4			
	15–20	5	71.4	2	38.6			
	> 20	3	60	2	40			

Nature of the farm	Urban	112	64.0	63	36	0.11	1	0.7
	Rural	4	57.1	3	42.9			

**Table 3:** Farming practice as predictor for calf morbidity.

Variable	Categories	Yes		No		Chi-square value	Df	*p* value
		Frequency	%	Frequency	%			
What types of feed container do you use?	Plastic	31	70.5	13	29.5	0.13	2	0.9
	Sack	70	71.4	28	70			
	Stainless steel	14	70	6	14			

What is the source of water?	Well	57	79.2	15	20.8	8.04	3	0.04
	River	12	70.6	5	29.4			
	Mixed sources	18	81.8	4	10.2			
	Tape water	40	58	29	42			

At what time interval of time do you clean your barn?	Once	16	61.5	10	16	3.24	4	0.5
	Twice	28	71.8	11	28			
	Three times	29	74.4	10	29			
	Four times	24	80	6	24			
	Others	29	65.9	15	29			

Do you disinfect your barn?	No	14	82.4	3	17.6	2.17	1	0.04
	Yes	112	69.1	50	30.9			

Do you separate your calves from adult cows?	No	90	75	30	25	3.81	1	0.01
	Yes	36	61	23	39			

Did sick calf/calves receive modern treatment?	Yes	88	84.6	16	15.4	0.67	1	0.6
	No	19	82.6	4	17.4			

If you use modern treatment, do your animals recover after treatment?	No	56	96.6	2	3.4	2.02	2	0.7
	Partial	9	100	0	0			
	Yes	19	57.6	14	42.4			

Do you practice navel treatment and disinfection?	No	90	75	30	25	0.00	1	1
	Yes	13	54.2	11	45.8			

Do your animals get regular treatment such as antibiotic therapy?	No	81	72.3	31	27.7	0.70	1	0.4
	Yes	45	67.2	22	32.8			

Do you vaccinate you cows regularly?	Yes	98	71	40	29	0.17	1	0.7
	No	28	68.3	13	31.7			

Do you have foot bath (foot disinfection pit) at farm gate?	No	121	69.9	52	30.1	0.47	1	0.6
	Yes	5	83.3	1	16.7			

Have you got any form of training on farm management?	Yes	111	71.6	44	28.4	0.16	1	0.8
	No	19	67.9	9	32.1			

Do you feed first colostrum to your calf?	Yes	130	71	53	29	—	1	—
	No	0	0	0	0			

At what time do you feed first colostrum?	6 and less than 6 h	130	71	53	29	—	1	—
	Greater than 6 h	0	0	0	0			

How many liters of colostrum do you feed your calves?	Less than 3 L	88	75.2	29	24.8	3.75	1	0.01
	Greater or equal to 3 L	42	63.6	24	36.4			

**Table 4:** Farming practice as predictor for calf mortality.

Variable	Categories	Yes		No		Chi-square value	Df	*p* value
		No	%	No	%			
What types of feed container do you use?	Plastic	31	60.8	20	39.2	0.67	2	0.9
	Sack	73	68.2	34	31.8			
	Stainless steel	13	52	12	48			

What is the source of water?	Well	48	65.8	25	34.2	5.95	3	0.01
	River	45	58.4	32	41.6			
	Mixed sources	18	81.8	4	18.2			
	Tape water	6	54.5	5	45.5			

At what time interval of time do you clean your barn?	Once	15	53.6	13	46.4	9.72	4	0.04
	Twice	28	71.8	11	28.2			
	Three times	25	62.5	15	37.5			
	Four times	25	83.3	5	16.7			
	Others	24	52.2	22	47.8			

Do you disinfect your barn?	No	12	70.6	5	29.4	0.24		0.7
	Yes	105	63.3	61	36.7			

Do you separate your calves from adult cows?	No	86	69.9	37	30.1	5.70	1	0.017
	Yes	31	51.7	29	48.3			

Did sick calf/calves receive modern treatment?	Yes	94	68.6	43	31.4	0.60	1	0.8
	No	24	51	23	49			

Do you practice navel treatment and disinfection?	No	103	64.8	56	35.2	0.27	1	0.6
	Yes	14	58.3	10	41.7			

Do your animals get regular treatment such as antibiotic therapy?	No	78	67.2	38	32.8	1.39	1	0.2
	Yes	39	58.2	28	41.8			

Do you vaccinate your cows regularly?	No	29	69.1	13	30.9	0.61	1	0.5
	Yes	88	62.4	53	37.6			

Do you have foot bath at farm gate?	Yes	7	100	0	0	3.63	1	0.09
	No	110	62.5	66	37.5			

Have you got any form of training on dairying?	Yes	99	63.9	56	36.1	0.03		1
	No	18	64.3	10	35.7			

Do you feed first colostrum to your calves?	Yes	117	63.9	66	30.1	—	1	—
	No	0	0	0	0			

At what time do you feed first colostrum?	Less than 6 h	117	63.9	66	30.1	—	1	—
	Greater than 6 h	0	0	0	0			

How many liters of colostrum do you feed your calves?	Less than 3 L	79	67.5	38	32.5	1.39	1	0.2
	Greater or equal to 3 L	38	57.6	28	42.4			

## Data Availability

The data that support the findings of this study are available from the corresponding author upon request.

## References

[1] Fao (2018). Shaping the Future of Livestock: Sustainably, Responsibly and Efficiently. *The 10^th^ Global Forum for Food and Agriculture (GFFA)*.

[2] Coppock D. L. (2016). Pastoral System Dynamics and Environmental Change on Ethiopia’s North-Central Borana Plateau—Influences of Livestock Development and Policy. *Springer Earth System Sciences*.

[3] Negassa A., Rashid S., Gebremedhin B. (2011). *Livestock Production and Marketing. Ethiopia Strategy Support Program II (ESSP II)*.

[4] Fao Strategies for Sustainable Animal Agriculture in Developing Countries.

[5] Bettencourt E. M. V., Tilman M., Narciso V., Carvalho M. L. D., Henriques P. D. d. S. (2015). The Livestock Roles in the Wellbeing of Rural Communities of Timor-Leste. *Revista de Economia e Sociologia Rural*.

[6] Csa (2019). Agricultural Sample Survey 2018/19 Report on Livestock and Livestock Characteristics (Private Peasant Holdings). *Central Statistics Authority Statistical Bulletin*.

[7] Solomon A., Workalemahu A., Jabbar M. A., Ahmed M. A., Hurrisa B. (2003). Livestock Marketing in Ethiopia: A Review of Structure, Performance and Development Options.

[8] Shapiro B. I., Gebru G., Desta S. (2015). Ethiopia Livestock Master Plan.

[9] Abebe G. Community–based Animal Health Services Delivery in Ethiopia. Experiences and the Way Forward on Community–based Animal Health Service Delivery in Ethiopia.

[10] Behnke R., Metaferia F. (2011). The Contribution of Livestock to Ethiopian Economy. *Livestock Policy Initiative*.

[11] World Bank (2011). Module 4-Smallholder Dairy Production.

[12] Agp (2013). Agricultural Growth Project for Livestock Market Development. *Value Chain Analysis for Ethiopia: Dairy, Meat and Live Animals and Hides, Skins and Leather*.

[13] Gebreyohanes G., Yilma Z., Moyo S., Mwai O. A. (2021). *Dairy Industry Development in Ethiopia: Current Status, Major Challenges and Potential Interventions for Improvement*.

[14] Staal S. J., Pratt A. N., Jabbar M. (2008). *Dairy Development for the Resource Poor A Comparison of Dairy Policies and Development in South Asia and East Africa A Conceptual Framework for Dairy Development*.

[15] Wudu T., Kelay B., Mekonnen H. M., Tesfu K. (2008). Calf Morbidity and Mortality in Smallholder Dairy Farms in Ada’a Liben District of Oromia, Ethiopia. *Tropical Animal Health and Production*.

[16] Gitau G. K., Aleri J. W., Mbuthia P. G., Mulei C. M. (2010). Causes of Calf Mortality in Peri-Urban Area of Nairobi, Kenya. *Tropical Animal Health and Production*.

[17] Razzaque M. A., Bedair M., Abbas S. (2009). Performance of Pre-weaned Female Calves Confined in Housing and Open Environment Hutches in Kuwait. *Pakistan Veterinary Journal*.

[18] Mellado M., Lopez E., Veliz F. G. (2014). Factors Associated with Neonatal Dairy Calf Mortality in a Hot-Arid Environment. *Livestock Science*.

[19] Svensson C. A., Linder A., Olsson S. O. (2006). Mortality in Swedish Dairy Calves and Replacement Heifers. *Journal of Dairy Science*.

[20] Chenyambuga S. W., Mseleko K. F. (2009). Reproductive and Lactation Performance of Ayshire and Boran Crossbred Cattle Kept in Smallholder Farms in Mufindi District, Tanzania. *Resea for Rural Developement*.

[21] Wymann M. N., Bonfoh B., Schelling E. (2006). Calf Mortality Rate and Causes of Death under Different Herd Management Systems in Peri-Urban Bamako, Mali. *Livestock Science*.

[22] Abdullatief E., Mansour M., Abdelgadir A. (2014). Major Causes and Risk Factors Associated with Calf Mortality in Dairy Farms in Khartoum State, Sudan. *Journal Medicine Animal Health*.

[23] Goshu G. (2017). Genetics of Threshold Characters and Distribution Cow Calving Traits in Holstein Friesian Cattle at Holeta Bull Dam Station, Ethiopia. *Journal of Veterinary Science & Technology*.

[24] Bekele M., Abduba Y., Alemayehu R., Fufa A., Kassahun A., Kebede A. (2009). Prevalence and Incidence Rates of Calf Morbidity and Mortality and Associated Risk Factors in Smallholder Dairy Farms in Hawassa, Southern Ethiopia. *Ethiopian Journal*.

[25] Ferede Y., Mazengia H., Bimrew T. (2014). Pre-weaning Morbidity and Mortality of Crossbred Calves in Bahir Dar Zuria and Gozamen Districts of Amhara Region, Northwest Ethiopia. *OALib*.

[26] Wold A. G., Yehualashet T. Note on Calf Mortality Rate at Two IAR Livestock Stations: Holetta and Adami Tulu.

[27] Klein-Jöbstl D., Iwersen M., Drillich M. (2014). Farm Characteristics and Calf Management Practices on Dairy Farms with and without Diarrhea: A Case–control Study to Investigate Risk Factors for Calf Diarrhea. *Journal of Dairy Science*.

[28] Olsson S. O., Viring S., Emanuelsson U., Jacobsson S. O. (1993). Calf Diseases and Mortality in Swedish Dairy Herds. *Acta Veterinaria Scandinavica*.

[29] Batiley A. (2018). Passive Immunity Status in New Born Calf under Pastoral Production System. *AAU Institutional Repository*.

[30] Assefa A., Ashenafi K. (2016). Dairy Calf Morbidity and Mortality and Associated Risk Factors in Sodo Town and its Suburbs, Wolaita Zone. *Ethiopia Journal Animal Science*.

[31] Tora E., Abayneh E., Seyoum W., Shurbe M. (2021). Longitudinal Study of Calf Morbidity and Mortality on Smallholder Farms in Southern Ethiopia. *PLoS One*.

[32] Eshetu G. (2014). Major Causes of Calf Mortality in Intensive Dairy Farms, Central Ethiopia: A Cohort Study. *International Journal of Livestock Research*.

[33] Fentie T., Guta S., Mekonen G. (2020). Assessment of Major Causes of Calf Mortality in Urban and Periurban Dairy Production System of Ethiopia. *Veterinary Medicine International*.

[34] Tora E., Shrube M., Kaba T., Seyoum W. (2021). Prevalence of Calf Mortality in Ethiopia: A Systematic Review and Meta-Analysis. *Veterinary Medicine International*.

[35] Ahmedin U. M., Assen A. A. (2023). Calf Morbidity, Mortality, and Management Practices in Dairy Farms in Jimma City, Southwestern Ethiopia. *BMC Veterinary Research*.

[36] Bard (2018). Tigray Region Bureau of Agriculture and Rural Development. Public Relation and Communication.

[37] Creswell J. W. (2009). *Research Design: Qualitative, Quantitative, and Mixed Methods Approaches*.

[38] Kothari C. R. (1978). *Quantitative Techniques*.

[39] Hennink M. M., Kaiser B. N., Weber M. B. (2019). What Influences Saturation? Estimating Sample Sizes in Focus Group Research. *Qualitative Health Research*.

[40] Muellmann S., Brand T., Jürgens D., Gansefort D., Zeeb H. (2021). How Many Key Informants Are Enough? Analysing the Validity of the Community Readiness Assessment. *BMC Research Notes*.

[41] Catley A. (2005). Participatory Epidemiology: A Guide for Trainers. *African Union/Intera-frican Bureau for Animal Resources*.

[42] Gebru G., Gebretinsae T. (2018). Evaluating the Implementation of Hazard Analysis Critical Control Point (HACCP) in Small Scale Abattoirs of Tigray Region, Ethiopia. *Food Protection Trends*.

[43] Graneheim U. H., Lundman B. (2004). Qualitative Content Analysis in Nursing Research: Concepts, Procedures and Measures to Achieve Trustworthiness. *Nurse Education Today*.

[44] Tesfaye T., Getachew S., Tadele A., Olbamo T. (2020). Study on the Causes of Calf Morbidity and Mortality and its Associated Risk Factors in South Omo Zone, South-Western Ethiopia. *Journal of Animal Science and Veterinary Medicine*.

[45] Alemu Y. F., Jemberu W. T., Mekuriaw Z., Abdi R. D. (2022). Incidence and Predictors of Calf Morbidity and Mortality from Birth to 6-months of Age in Dairy Farms of Northwestern Ethiopia. *Frontiers in Veterinary Science*.

[46] Abebe R., Dema T., Libiyos Y. (2023). Longitudinal Study of Calf Morbidity and Mortality and the Associated Risk Factors on Urban and Peri-Urban Dairy Farms in Southern Ethiopia. *BMC Veterinary Research*.

[47] Asmare A. A., Kiros W. A. (2016). Dairy Calf Morbidity and Mortality and Associated Risk Factors in Sodo Town and its Suburbs, Wolaita Zone, Ethiopia. *Slovak Journal of Animal Science*.

[48] Dagne K., Kassa T., Kebede N. (2018). Occurrences of Dairy Calf Mortality and Morbidity and the Associated Risk Factors in Sululta and its Environs, Central Ethiopia. *International Journal of Veterinary Sciences and Animal Husbandry*.

[49] Mohammed R., Kefyalew H., Kassaye D. (2020). Incidence of Calf Morbidity and its Predictors in North Shewa, Amhara, Ethiopia. *Veterinary Medicine International*.

[50] Gulliksen S. m., Lie K. I., Løken T., Østerås O. (2009). Calf Mortality in Norwegian Dairy Herds. *Journal of Dairy Science*.

[51] Belay T., Mekibib B. (2022). Assessment of Calf Management and Hygiene Practices Adopted in Large and Small-Scale Dairy Farms in Wondo Genet Area, Southern Ethiopia. *Veterinary Medicine: Research and Reports*.

[52] Yitagesu E., Fentie T., Kebede N., Jackson W., Smith W. (2022). The Magnitude of Calf Morbidity and Mortality and Risk Factors in Smallholder Farms across Livestock Production Systems in Central Ethiopia. *Veterinary Medicine and Science*.

[53] Weaver D. M., Tyler J. W., VanMetre D. C., Hostetler D. E., Barrington G. M. (2000). Passive Transfer of Colostral Immunoglobulins in Calves. *Journal of Veterinary Internal Medicine*.

[54] Uetake K. (2013). Newborn Calf Welfare: A Review Focusing on Mortality Rates. *Animal Science Journal*.

[55] Deddefo A., Mamo G., Asfaw M., Amenu K. (2023). Factors Affecting the Microbiological Quality and Contamination of Farm Bulk Milk by Staphylococcus aureus in Dairy Farms in Asella, Ethiopia. *BMC Microbiology*.

[56] Fereja A. B., Aboretugn N. F., Bulti N. Q. (2023). Determination of Microbial Hygiene Indicators of Raw Cow Milk in Assosa District, Ethiopia. *Journal of Food Quality*.

